# Coenzyme Q_10_ protected against arsenite and enhanced the capacity of 2,3-dimercaptosuccinic acid to ameliorate arsenite-induced toxicity in mice

**DOI:** 10.1186/s40360-021-00484-z

**Published:** 2021-04-07

**Authors:** Victoria K. Mwaeni, James N. Nyariki, Ngalla Jillani, George Omwenga, Mathew Ngugi, Alfred Orina Isaac

**Affiliations:** 1 Department of Biochemistry and Biotechnology, Technical University of Kenya, P. O. Box 52428, Nairobi, 00200 Kenya; 2 Institute of Primate Research, P.O. Box 24481, Karen, Nairobi, 00502 Kenya; 3 Department of Biochemistry, Microbiology and Biotechnology, Kenyatta University, P.O. Box 43844-00100, Nairobi, Kenya; 4 Department of Pharmaceutical Sciences and Technology, Technical University of Kenya, P. O. Box 52428, Nairobi, 00200 Kenya

**Keywords:** Ubiquinone, Organ metal damage, Glutathione, Inflammation, Immune response

## Abstract

**Background:**

Arsenic poisoning affects millions of people. The inorganic forms of arsenic are more toxic. Treatment for arsenic poisoning relies on chelation of extracellularly circulating arsenic molecules by 2,3-dimecaptosuccinic acid (DMSA). As a pharmacological intervention, DMSA is unable to chelate arsenic molecules from intracellular spaces. The consequence is continued toxicity and cell damage in the presence of DMSA. A two-pronged approach that removes extracellular arsenic, while protecting from the intracellular arsenic would provide a better pharmacotherapeutic outcome. In this study, Coenzyme Q_10_ (CoQ_10_), which has been shown to protect from intracellular organic arsenic, was administered separately or with DMSA; following oral exposure to sodium meta-arsenite (NaAsO_2_) – a very toxic trivalent form of inorganic arsenic. The aim was to determine if CoQ_10_ alone or when co-administered with DMSA would nullify arsenite-induced toxicity in mice.

**Methods:**

Group one represented the control; the second group was treated with NaAsO_2_ (15 mg/kg) daily for 30 days, the third, fourth and fifth groups of mice were given NaAsO_2_ and treated with 200 mg/kg CoQ_10_ (30 days) and 50 mg/kg DMSA (5 days) either alone or in combination.

**Results:**

Administration of CoQ_10_ and DMSA resulted in protection from arsenic-induced suppression of RBCs, haematocrit and hemoglobin levels. CoQ_10_ and DMSA protected from arsenic-induced alteration of WBCs, basophils, neutrophils, monocytes, eosinophils and platelets. Arsenite-induced dyslipidemia was nullified by administration of CoQ_10_ alone or in combination with DMSA. Arsenite induced a drastic depletion of the liver and brain GSH; that was significantly blocked by CoQ_10_ and DMSA alone or in combination. Exposure to arsenite resulted in significant elevation of liver and kidney damage markers. The histological analysis of respective organs confirmed arsenic-induced organ damage, which was ameliorated by CoQ_10_ alone or when co-administered with DMSA. When administered alone, DMSA did not prevent arsenic-driven tissue damage.

**Conclusions:**

Findings from this study demonstrate that CoQ_10_ and DMSA separately or in a combination, significantly protect against arsenic-driven toxicity in mice. It is evident that with further pre-clinical and clinical studies, an adjunct therapy that incorporates CoQ_10_ alongside DMSA may find applications in nullifying arsenic-driven toxicity.

## Introduction

About 140 million people in more than 50 countries drink underground water with high arsenic levels (> 10 mg/l) [1]. There is a tremendous gap in research on the negative effects of arsenic on normal human development and how some of these profound adverse effects can be mitigated. Environmental forms of arsenic include methylarsenic acid (MMA), dimethylarsinic acid (DMAA), trimethylarsine oxide (TMAO), arsenite, arsenate, arsenous acids and arsenic acids [2]. Arsenite and arsenate are the most common forms of arsenic. Arsenite is considered as the most toxic form due to its high affinity for sulfhydryl groups in proteins and enzymes that are vital in cell signalling and metabolic processes [3]. Depending on the level and duration of exposure, arsenic can result in acute and chronic toxicity.

Upon absorption, arsenic undergoes methylation, generating toxic intermediates with concomitant utilization of glutathione (GSH) as a cofactor [4–6]. Such utilization of endogenous GSH depletes its levels. As a potent antioxidant, the depletion of GSH triggers accumulation of lethal reactive oxygen species (ROS), consequently resulting in oxidative stress. Previous studies have demonstrated clear evidence for arsenic-induced depletion of other vital antioxidants such as catalase, superoxide dismutase, and glutathione peroxidase [7]. Oxidative injury as a result of arsenic exposure contributes to inflammation and immune system disturbances linked to cardiovascular and kidney disease, as well as cancer [8–10]. Generation of reactive oxygen species has been implicated in arsenic-induced anemia as a result of lipid peroxidation of the erythrocytic membrane and inhibition of the erythogenin protein, compromising erythropoiesis [11]. The apoptotic-inducing effect of arsenic caused by activation of caspases and increased ROS production is associated with other effects such as alteration of granulocyte levels, neurotoxicity, hepatotoxicity and renal damage [12]. Consequently, arsenic poisoning is associated with severe organ damage, hematopoietic abnormalities, inflammation, oxidative stress, DNA damage and nervous system degeneration [13]. The reported toxicological profile demonstrate the importance of controlling oxidative stress and inflammation in arsenic toxicity.

From the literature review, it is incredibly clear that inorganic arsenic wreaks havoc to various physiological and biochemical processes. The most affected are those that protect from oxidative stress and inflammation, with enormous implications in hematopoiesis and immune function. Current treatment options rely on chelation therapy, which is deficient due to inability to remove arsenic from intracellular spaces. The result is continued cellular damage during and after chelation therapy. Dimercaprol (BAL) was the first chelator used to treat arsenic exposure. It was later replaced by its analogs 2,3-dimercaptopropanesulphonate sodium (DMPS) and meso-2,3-dimercaptosuccinic acid (DMSA) due to their low toxicity and the ability to be delivered both orally and intravenously [14]. DMSA has been reported to promote extracellular distribution, neutropenia, and renal toxicity due to efflux of high levels of arsenic through the renal tubules [15]. The biggest limitation to DMSA is its inability to eliminate arsenic from the intracellular sites of cells due to its lipophobic nature [16]. It is evident that a treatment strategy that utilizes DMSA with other agents capable of blocking toxicity due to arsenic in intracellular spaces provides an opportunity for achieving full biochemical recovery in arsenic-induced toxicity. This is the hypothesis that formed the basis for this study.

The current study determined the ability of Coenzyme Q_10_ (CoQ_10_) and MDSA to protect from the toxic effects of an inorganic arsenic – sodium meta-arsenite (NaAsO_2_). CoQ_10_ has been shown to protect from organic forms of arsenic in drugs (melasoprol) [17]. CoQ_10_ is an important cofactor involved in the mitochondrial electron transport chain. Other studies have explored the importance of CoQ_10_ in attenuation of other heavy metal toxicities due to its antioxidant, anti-inflammatory and anti-apoptotic properties [18–21]. In a previous study, a combination of CoQ_10_ and vitamin E significantly prevented GSH depletion in arsenic toxicity [22]. The previously demonstrated protective effects of CoQ_10_ against organic forms of arsenic laid the foundation for the current study. Findings from this study demonstrate that CoQ_10_ and DMSA, individually and when combined, robustly assuaged arsenic-induced oxidative stress, inflammation and organ damage; lending credence to the hypothesis that CoQ_10_ may enhance attenuation of arsenic toxicity by DMSA.

## Materials and methods

### Animals and ethical statement

The present study involved use of mice and was performed under strict application of the 3R rules and the ARRIVE checklist for reporting animal research [23]. All experimental procedures and protocols involving mice in this study were approved by Institutional review for approval Committee (IRC) of the Institute of Primate Research Karen, Kenya (ISERC/08/2017). Accordingly, all experiments were conducted in compliance with the recommendations of the Helsinki declaration on guiding principles on care and use of animals. All research staff were trained prior to the beginning of the study in conformity to FELASA B guidelines and regulations. Male 4–5 weeks old Swiss White mice (weighing 22–25 g) were purchased from Kenya Medical Research Institute (KEMRI). The animals were housed in standard mice cages under controlled room temperature (25 °C) and a 12 h light/dark cycle. The animals were fed on mice pellets (Unga Feeds) and were provided with ad libitum access to water. The animals were allowed to acclimatize for 2 weeks before commencement of experimental procedures. Mice were allocated randomly to cages with *n* = 10 mice per group in concert with study objective and individual experimental groups. Humane endpoints like unarousable coma, retinal haemorrhages, dysconjugate gaze, pouting, decerebrate rigidity, respiratory distress and convulsions prior or/ and after treatment with CoQ10, DMSA and arsenite were monitored to limit suffering. In this study treatment and evaluation of the health status of mice were performed sequentially. Amidst these, no animals reached the criteria for humane endpoints or died. At the end of the experimental period (30 days post treatment), for ex vivo analysis mice were euthanised using Rompun (2%) and ketamine (50 mg/ml) through intra-muscular injection followed by perfusion with sterile PBS.

### Experimental design

Mice were randomly grouped into five groups of 10 mice each (*n* = 10 per group). Group 1 served as the normal control; Group 2 mice were treated with 15 mg/kg arsenite for 30 days; Group 3 mice received 200 mg/kg of CoQ_10_ first for 15 days and then continued simultaneously with 15 mg/kg arsenite for another 30 days; Group 4 mice were administered with 15 mg/kg arsenite for 30 days and then treated with 50 mg/kg DMSA for 5 days from the 25th days post treatment and Group 5 received 15 mg/kg arsenite and CoQ_10_ (200 mg/kg) for 30 days and then treated with DMSA (50 mg/kg for 5 days from 25th days post treatment). A dose of 200 mg/kg CoQ_10_ was selected for this study based on a previous finding that 200 mg/kg CoQ_10_ is sufficient to elevate brain mitochondrial concentration and further provide neuroprotection to mice [24]. Besides, the effectiveness of 200 mg/kg CoQ_10_ as an anti-inflammatory and anti-oxidant agent has been demonstrated [25]. A dose of 15 mg/kg arsenite was chosen for this study based on the findings that 15 mg/kg is able to induce toxicity in wild type mice characterized by vascular permeability and increasing arsenic concentration in blood and tissues [26, 27]. A dose of 50 mg/kg for 5 days was administered to mice exposed to sodium arsenite. Studies have shown that 50 mg/kg DMSA for 5 days is effective in enhancing renal excretion and decreases blood concentration of heavy metals [28–31]. All the treatments were administered orally by the use of a gavage.

### Preparation of coenzyme Q_10,_ sodium arsenite and DMSA

*CoQ*_*10*_ *powder (98%),* sodium meta-arsenite (Trivalent-inorganic arsenic) and Meso-2,3-Dimercaptosuccinic Acid (98%) *were purchased from* Sigma Aldrich (St Louis, MO). *CoQ*_*10*_ *was* prepared by dissolving it in olive oil solution. On the other hand sodium meta-arsenite and Meso-2,3-Dimercaptosuccinic Acid were prepared by dissolving them in deionized water.

### Sample collection

Blood samples were obtained through cardiac puncture and used for hematology and serum analyses. Blood samples for hemogram analysis were collected in EDTA tubes. For serum analysis, blood samples were left to settle at room temperature for 1 hour and centrifuged at 10000 rpm at 4 °C for 5mins (Centurion Scientific Ltd. K240R, UK).

Liver, kidney, and brain samples were harvested and used for GSH and histological analysis. For Histology, organ samples were weighed and fixed using 4% formalin and stored at room temperature. For GSH assay, the organs were weighed and homogenized using homogenization buffer (0.25 M sucrose, 5 Mm Hepes-Tris pH 7.4 with protease inhibitor). The homogenates were aliquoted into cyrovial tubes and stored at -80 °C.

### Determination of weight

The weights of the mice were taken using an analytical balance (Mettler PM34, DoltaRange®) at the beginning (day 0) and at the end of the experiment (day 45 post-treatment) to determine weight change during the period of study.

### Hematology and biochemical analysis

Blood samples were subjected to full hemogram analysis using an automated analyzer (Sysmex XS 1000i Hematology Analyzer, WA, USA). Serum Triglycerides, High Density Lipoprotein Cholesterol, cholesterol, Aspartate aminotransferase (AST), Alanine aminotransferase (ALT), Gamma-glutamyl transferase (GGT), total bilirubin, and creatinine levels were determined using an automated analyzer (Integra-400 plus analyzer, Roche, Basel, Switzerland).

### Cytokine ELISA

Serum was also used to determine the levels of pro-inflammatory cytokines (IFN-γ and TNF α), and anti-inflammatory cytokine (Interleukin 10) using cytokine-specific sandwich ELISA kit (Invitrogen, Thermo Fischer Scientific, California, USA) following the manufacturer’s instructions. ELISA plate reader set at an absorbance of 450 nm was used for measurement of the cytokine concentration.

### Assessment for GSH in liver, kidney and brain samples

Glutathione assay was performed as reported by Rahman, et al., [32] with slight modifications. GSH standard of 200 μmol/l was prepared in 0.5% sulphosalicylic acid (SSA). Two-fold serial dilution of 0.5% SSA was made as follows; 100, 50, 25, 12.5, 6.25, 3.13 and 1.56 μmol/l. The substrate, 5,5′-dithiobis (2-nitrobenzoic acid (DTNB), was prepared using 0.1 M potassium phosphate and 5 mM EDTA disodium salt, pH 7.5) (KPE buffer) to 0.6 mg/ml. A volume of 50 μl of brain/liver/kidney tissue homogenates was mixed with 50 μl sulphosalicylic acid (5% w/v) and 0.25 mM EDTA and centrifuged at 10000 rpm. A volume of 25 μl of GSH standard was then loaded in the first two columns of a 96-well microtitre plate, specifically wells B–H. A volume of 25 μl of the sample was loaded to the remaining wells. One hundred microliter of the substrate (DNTB) was then added to each well and absorbance was measured at 405 nm using a multi-detection microtitre plate reader (R&D Systems, Minneapolis, MN).

### Histopathological examination of liver, kidney and brain samples

The organ samples fixed in 4% formalin were processed by dehydration with ethanol, and embedded using paraffin wax. Sections of 5 μm thickness were cut and mounted on glass slides. Hematoxylin and eosin stain was used for staining and sections examined using a microscope (Zeis Axio scope).

### Statistical analysis

Statistical analysis was performed using one-way ANOVA with Tukey’s post hoc test for multiple comparisons with the level of significance set at *p* < 0.05. Analysis was done using Graph Pad Prism Software Version 5.0 (San Diego, CA).

## Results

### Effects of Arsenite, CoQ_10_ and DMSA on weight

**Fig. 1:**
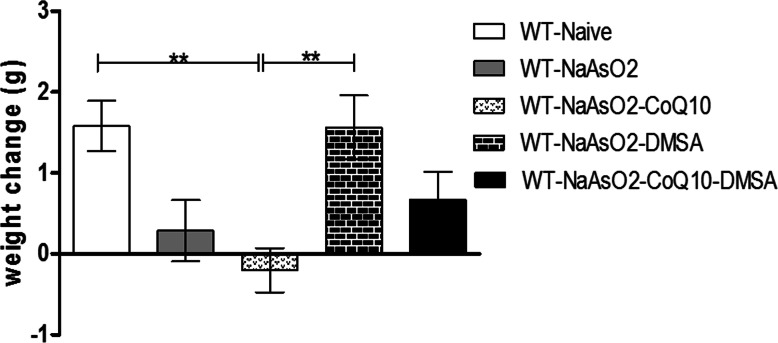
The figure shows the effects of arsenite, CoQ_10_ and DMSA on weight. Mice were administered with 15 mg/kg arsenite alone or alongside 200 mg/kg of CoQ_10_ or 50 mg/kg DMSA independently or in combination. Initial body weight was determined. The final weight was determined after 45 days’ post treatment. Change in weight was calculated and analysed using one-way ANOVA with Tukey’s test for group comparisons. Bars represent ± SEM. The indicated level of significance **p < 0.01. *n* = 10 mice

Oral exposure to arsenite induced a reduction in weight when compared to the normal control group (Fig. 1). The CoQ_10_ group showed a significant drop in weight (*p* < 0.01) when compared to the normal control and the arsenite group. CoQ_10_ did not protect from arsenite-induced weight loss. Notably, administration of DMSA ameliorated arsenite-driven weight loss (Fig. 1).

### CoQ_10_ and DMSA assuaged arsenite-driven impact on the hematocrit, RBC and hemoglobin levels

**Fig. 2:**
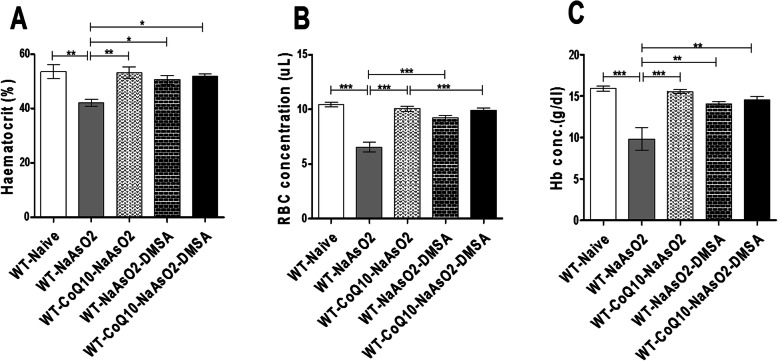
The figure shows the effects of arsenite, CoQ10 and DMSA on the hematocrit, RBCs, and hemoglobin. Male Swiss mice were administered with 15 mg/kg arsenite alone and others were treated with 50 mg/kg DMSA and 200 mg/kg CoQ_10_ independently or in combination. Mice were sacrificed after 45 days of treatment and blood obtained. RBC Count (**a**), and hemoglobin content (**b**) were determined using the hemogram autoanalyser. Analysis was done using one-way ANOVA with Tukeys test for comparison of group means. Bars represent ± SEM Statistical significance key: *p < 0.05,**p < 0.01, ***p < 0.001 *n* = 10 mice per group

Next we determined whether DMSA and CoQ_10_ alone or in combination could block arsenite-induced negative effects on the hematocrit values, RBC counts, and hemoglobin levels. Exposure to arsenite significantly depleted (*P* < 0.01) the haematocrit levels when compared to the control group. Treatment with CoQ_10_ or DMSA separately or when combined with DMSA significantly protected (*P* < 0.05) from arsenite-induced suppression of the haematocrit (Fig. 2a).

Further, exposure to arsenite led to a significant decrease (*p* < 0.001) in the RBC count (Fig. 2b) and hemoglobin concentration (Fig. 2c).

### DMSA and CoQ_10_ ameliorated arsenic driven negative effects on RBC indices

**Fig. 3:**
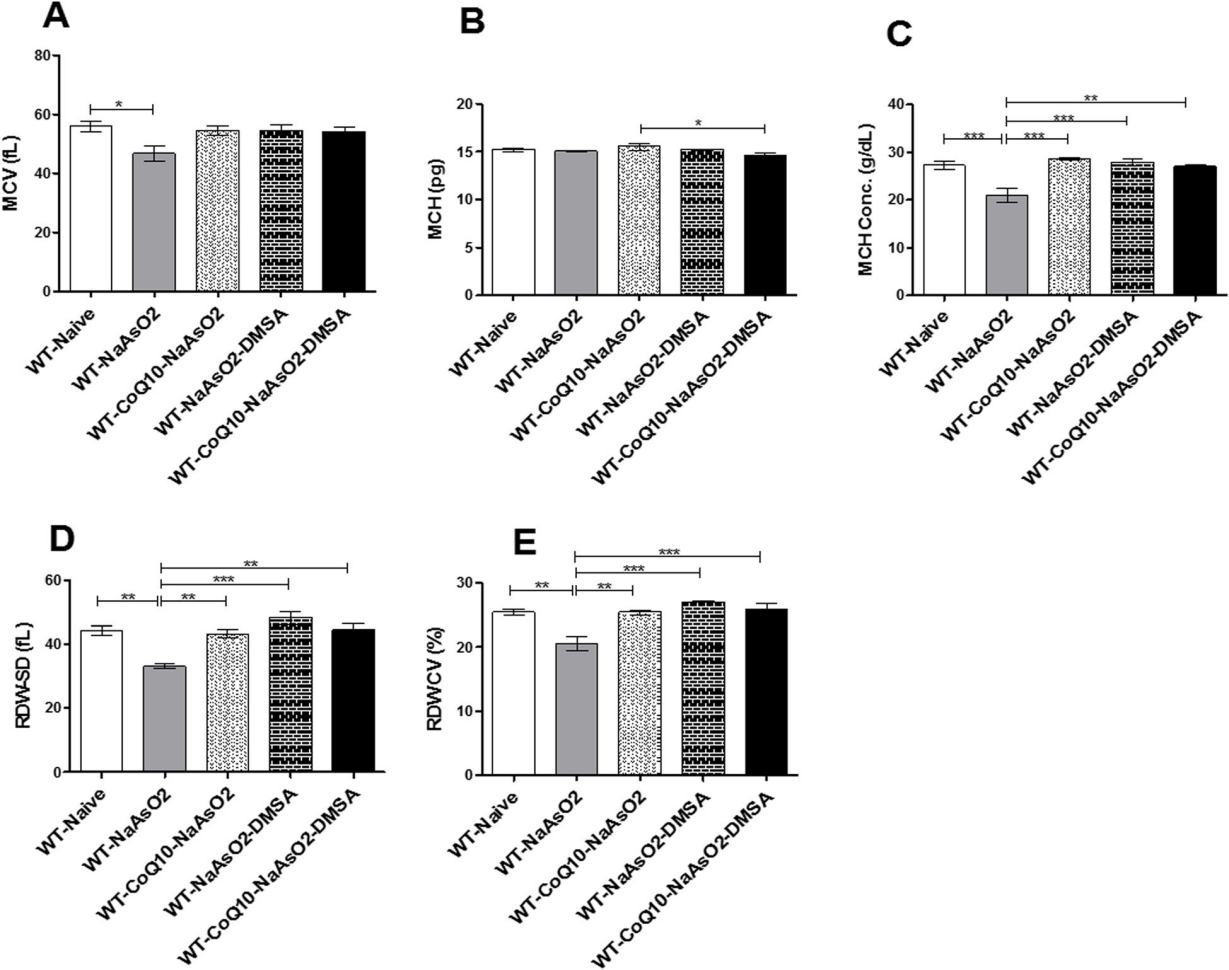
The effect of arsenite, CoQ_10_, and DMSA on various red blood cell indices. Male Swiss mice were treated with 15 mg/kg arsenite daily for 45 days. Two groups of mice were receiving 50 mg/kg DMSA or 200 mg/kg CoQ_10_ alone while another group of mice were treated using combination of the two. After the 45 days post treatment, blood was obtained by cardiac puncture and RBC indices determined include MCV (**a**), MCH (**b**), MCHC (**c**), RDW-SD (**d**), and RDW-CV (**e**) using blood cell auto-analyzer. One-way ANOVA was used for analysis and Tukey test followed for multiple comparisons. Bars represent ± SEM Significance difference *p < 0.05**p < 0.01, ***p < 0.001 *n* = 10

Arsenite-induced a significant decrease (*p* < 0.05) in mean corpuscular volume (MCV) (Fig. 3a). Administration of DMSA and CoQ_10_ separately or in combination blocked the arsenite-induced decrease in MCV. Arsenite, DMSA and CoQ_10_ did not have any effect on the mean corpuscular hemoglobin (MCH) (Fig. 3d). However, Arsenite resulted in significant (*p* < 0.001) depletion of the mean corpuscular hemoglobin concentration (MCHC) (Fig. 3c). Treatment with DMSA and CoQ_10_ either alone or when combined, significantly prevented arsenite-induced decrease of MCHC.

Exposure to arsenite resulted in significantly low (*p* < 0.001) red distribution width-standard deviation (RDW-SD) in comparison with the control group (Fig. 3d). Oral administration of CoQ_10_ alone or when combined with DMSA mitigated the arsenite-induced decrease in RDW-SD and RDW-CV (Fig. 3e).

Overall, arsenite supressed all the RBC indices (MCV, MCHC, RDW-SD and RDW-CV), with the exception of the MCH (Fig. 3b). Notably, CoQ_10_ alone and when combined with DMSA nullified the negative effects of arsenite on the RBC indices.

### The effect of arsenite, CoQ_10_ and DMSA on WBCs and its subtypes

**Fig. 4:**
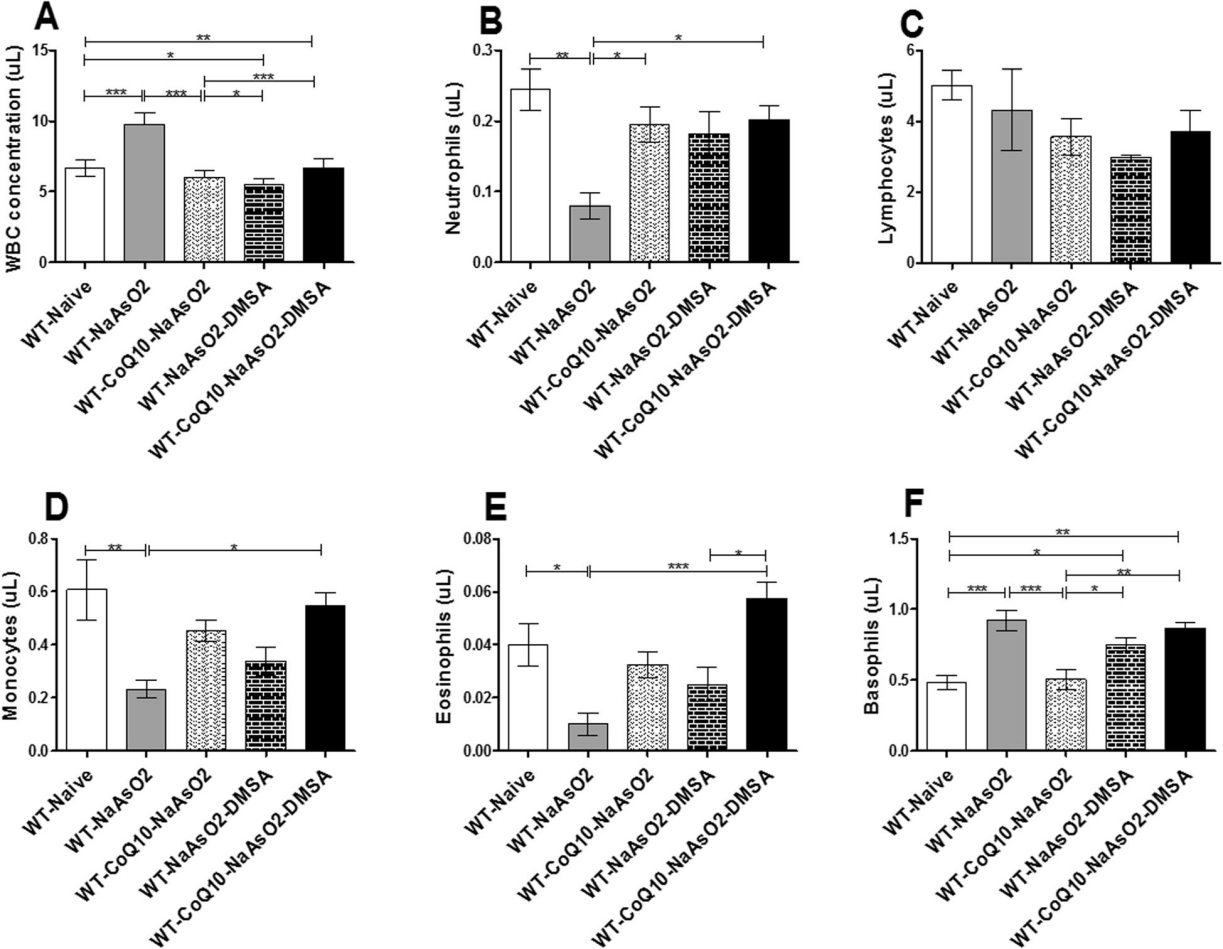
The effect of arsenite, CoQ_10_ and DMSA WBCs (**a**), neutrophils (**b**), monocytes (**c**), eosinophils (**d**), and basophils (**e**). Male Swiss mice were treated with 15 mg/kg arsenite daily for 45 days. Two groups of mice were receiving 50 mg/kg DMSA or 200 mg/kg CoQ_10_ alone while another group of mice were treated using combination of the two. After the 45 days post treatment, blood was obtained by cardiac puncture and the level of Total WBC and its subtypes was determined using blood cell autoanalyser. One-way ANOVA was used for analysis and Tukey test followed for multiple comparisons. Bars represent ± SEM. Significance difference *p < 0.05, **p < 0.01, ***p < 0.001. *n* = 10 mice

On 45 days’ post-treatment, mice exposed to arsenite alone showed significant (*P* < 0.001) elevation of WBC count when compared to the normal control group (Fig. 4a). Treatment with CoQ_10_ and DMSA significantly blocked the arsenite-driven elevation of WBC (Fig. 4a).

Arsenite administration resulted in a significant decrease (*P* < 0.01) in neutrophil levels. Treatment with CoQ_10_ alone or in combination with DMSA significantly (*P* < 0.05) prevented neutrophil suppression by arsenite (Fig. 4b).

Notably, arsenite resulted in statistically significant (*p* < 0.01) suppression of monocytes. The co-administration of CoQ_10_ and DMSA nullified the arsenite-induced monocyte suppression (Fig. 4c). The eosinophil levels were significantly reduced (*p* < 0.05) in mice exposed to arsenite when compared to the control group. However, the combined treatments of CoQ_10_ and DMSA significantly protected from arsenite-induced suppression of eosinophils (Fig. 4d). Arsenite resulted in elevation of basophils when compared with the control group. Administration of CoQ_10_ significantly (*P* < 0.001) stabilized basophils levels in the presence of arsenite. Once again, treatment with DMSA and CoQ_10_ blocked arsenite-induced basophil elevation (Fig. 4e). It’s important to note that CoQ_10_ was more effective in nullifying the negative effects due arsenite in regard to basophils as compared to DMSA alone or when combined with CoQ_10_. Administration of arsenite, CoQ_10_ or DMSA did not have a statistically significant effect on lymphocyte levels (Fig. 4f).

### Effects of arsenite, CoQ_10_ and DMSA on platelets

**Fig. 5:**
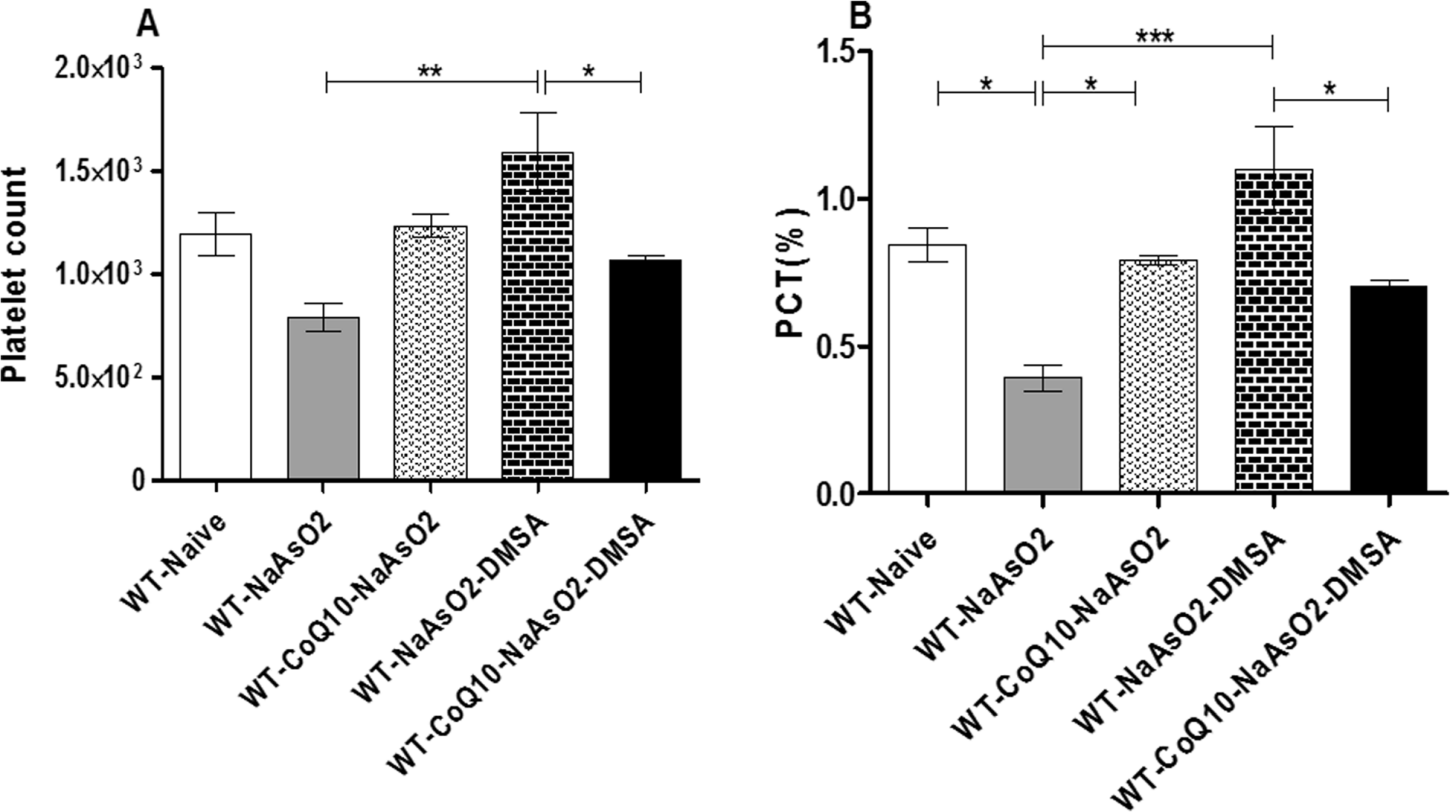
The figure shows the effect of arsenite, CoQ_10_ and DMSA on platelets. Male Swiss mice were treated with 15 mg/kg arsenite daily for 45 days. Two groups of mice were receiving 50 mg/kg DMSA or 200 mg/kg CoQ_10_ alone while another group of mice were treated using combination of the two. After the 45 days post treatment, blood was obtained by cardiac puncture and the level of platelets (**a**) and platelecrit (**b**) determined. One-way ANOVA was used for analysis and Tukey test followed for multiple comparisons. Bars represent ± SEM. Indicated level of significance: *p < 0.05, **p < 0.01, ***p < 0.001. *n* = 10 mice per group

Arsenic suppressed the total platelet count when compared to the control group. Administration of CoQ_10_ ameliorated the arsenite-induced depletion of platelets (Fig. 5a). Additionally, a significant (*P* < 0.05) arsenite-induced reduction of the platelecrit was noted. However, CoQ_10_ assuaged the arsenite-driven suppression of the platelets (Fig. 5b). Mice treated with DMSA alone recorded a significantly higher PLT (Fig. 5a) and PCT (Fig. 5b) when compared with the co-administered group (CoQ_10_ + DMSA). In this regard, CoQ_10_ was very potent in stabilizing platelet/platelecrit levels in the presence of the toxicant – arsenite. In summary, CoQ_10_ mitigated arsenite-driven thrombocytopenia when administered alone. In the presence of arsenic, DMSA clearly induced thrombocytosis (high platelet count). This DMSA-driven effect was eliminated in the co-administered group (CoQ_10_ + DMSA).

### The effect of arsenite, CoQ_10_ and DMSA on cholesterol, triglycerides and HDLC levels

**Fig. 6:**
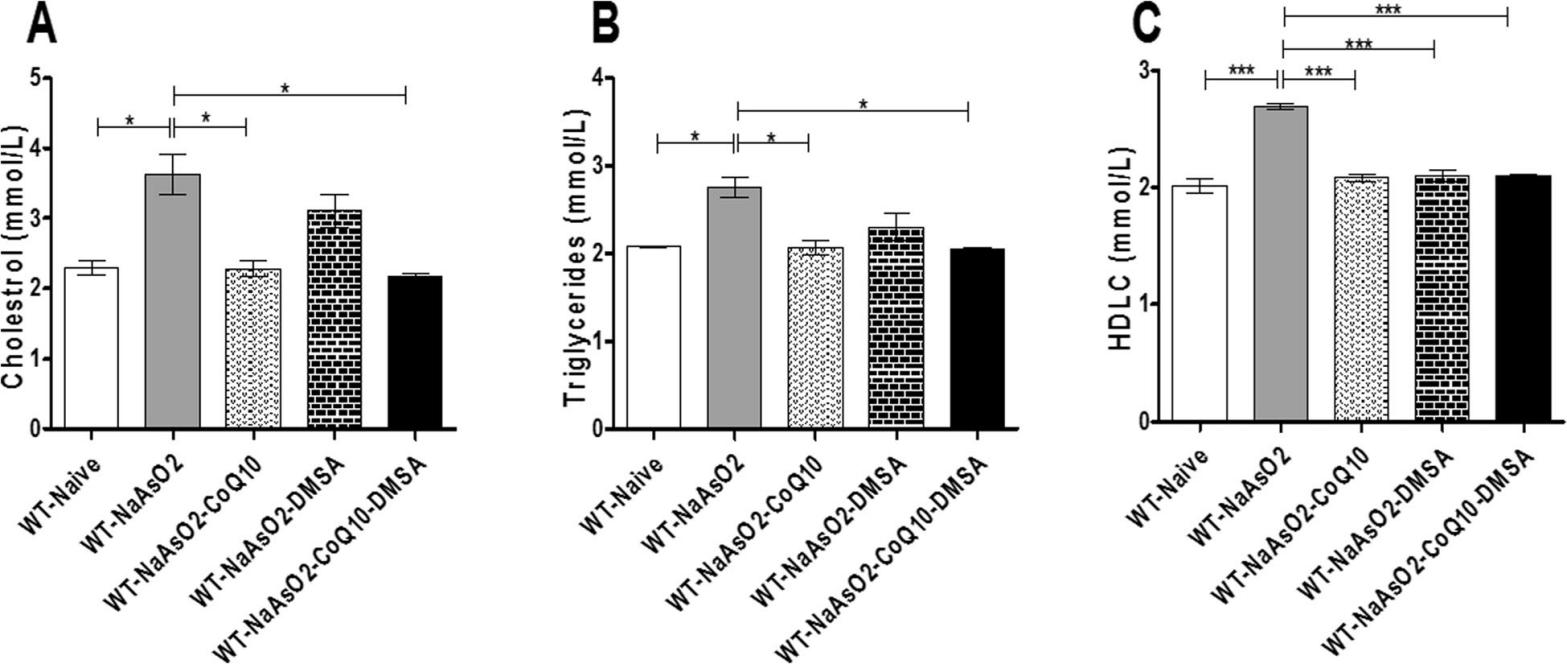
The figure shows the effects of arsenite, CoQ_10_ and DMSA on the lipid profile. Mice were administered with 15 mg/kg arsenite alone or alongside 200 mg/kg of CoQ_10_ or 50 mg/kg DMSA independently or in combination. After 45 days’ post treatment, whole blood was obtained followed by serum harvesting. Serum obtained was used in determining the levels of cholesterol (**a**), Triglycerides (**b**) and HDLC (**c**). One-way ANOVA was used for analysis and Tukey test followed for multiple comparisons. Bars represent ± SEM. Level of significance: *p < 0.05, ***p < 0.001. *n* = 10 mice per group

Administration of sodium arsenite resulted in a significant (*p* < 0.05), elevation of cholesterol (Fig. 6a) triglycerides (Fig. 6b), and HDLC (Fig. 6c). Treatment with CoQ_10_ alone and when combined with DMSA significantly ameliorated the arsenic-driven elevation of cholesterol, triglycerides and HDLC. It was further noted that CoQ_10_ was more effective in mitigating arsenite-induced elevation of cholesterol when compared with DMSA (Fig. 6a and b).

### The effects of arsenite*,* CoQ_10_ and DMSA on GSH levels in the liver, brain and kidney tissues

**Fig. 7:**
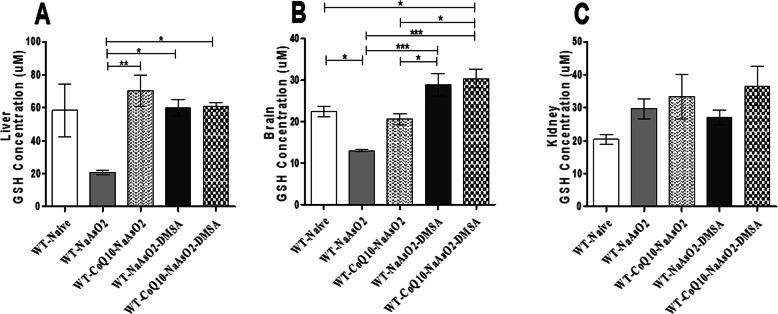
The figure shows the effect of arsenite, CoQ_10_ and DMSA on GSH levels in the liver, brain and kidney. Mice were administered with 15 mg/kg arsenite alone or alongside 200 mg/kg of CoQ_10_ or 50 mg/kg DMSA independently or in combination After 45 days’ post treatment, mice were sacrificed and organs harvested. The GSH levels were determined in the liver (**a**), brain (**b**), and kidney (**c**). Concentrations obtained were analyzed using one-way ANOVA with Tukey’s test for group comparisons. Bars represent ± SEM. The level of significance was set as follows; *p < 0.05, **p < 0.01, ***p < 0.001, *n* = 10

Exposure to arsenite resulted in a concomitant depletion of GSH in the liver and brain (Fig. 7a, b). Treatment with CoQ_10_ and DMSA separately and when combined, significantly restored the GSH levels in the liver and brain tissue in the presence of the arsenite (Fig. 7a & b). Notably, in the presence of DMSA, the GSH elevation was highly significant, rising above the normal control for the brain. In stark contrast, GSH levels in the kidney were not statistically different from the normal control for mice administered with arsenite, CoQ_10_ and DMSA (Fig. 7c).

### The effects of sodium arsenite on liver function and ameliorative impact of CoQ_10_ and DMSA

**Fig. 8:**
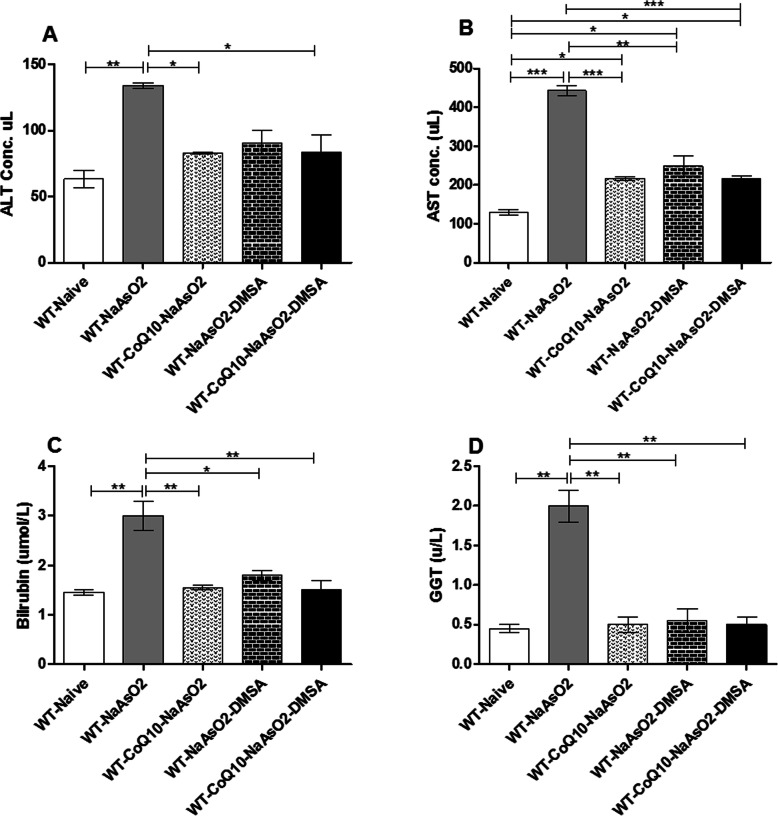
The figure shows the effect of arsenite, CoQ_10_ and DMSA on liver enzymes. Mice were administered with 15 mg/kg arsenite alone or alongside 200 mg/kg of CoQ_10_ or 50 mg/kg DMSA independently or in combination. After 45 days’ treatment period, the mice were sacrificed and blood obtained through cardiac puncture. Serum was then obtained and used for determination of ALT (**a**), AST (**b**), Bilirubin (**c**) and GGT (**d**) levels. Data was analyzed using one-way ANOVA and Tukey’s tests. Bars represent ± SEM. Levels of significance (*p < 0.05, **p < 0.01, ***p < 0.001). *n* = 10

Results from this study showed that arsenite induced significant elevation (*p* < 0.01) of ALT (Fig. 8a), AST (Fig. 8b), total bilirubin (Fig. 8c) and GGT (Fig. 8d). Notably, CoQ_10_ and DMSA separately or when combined ameliorated the arsenite-induced elevation of ALT, AST, bilirubin and GGT; a clear demonstration of a protective role.

### Sodium arsenite induced an increase in serum creatinine levels

**Fig. 9:**
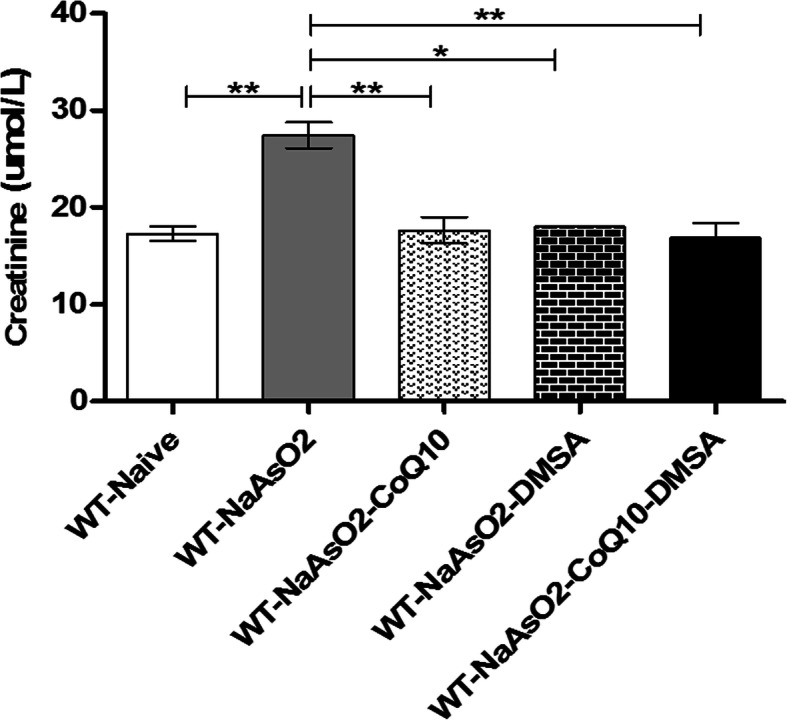
The figure shows the effect of arsenite, CoQ_10_ and DMSA on kidney tissue. Mice in the indicated group were treated with 15 mg/kg arsenite 200 mg/kg CoQ_10_, and 50 mg/kg DMSA. After 45-day treatment period, they were sacrificed, and blood obtained. Serum was processed and used to determine the creatinine levels. Data was analyzed using one-way ANOVA and Tukey’s test for group comparisons. Bars represent ± SEM. Level of significance *p < 0.05, **p < 0.01. n = 10 mice

Creatinine levels were significantly (*p* < 0.01) elevated in mice exposed to arsenite compared to the control group. Oral administration of CoQ_10_ and DMSA separately and when combined resulted in a significant *p* < 0.01) stabilization of serum creatinine levels in the presence of arsenite (Fig. 9).

### CoQ_10_ supplementation modulated arsenic-induced inflammatory response

**Fig. 10:**
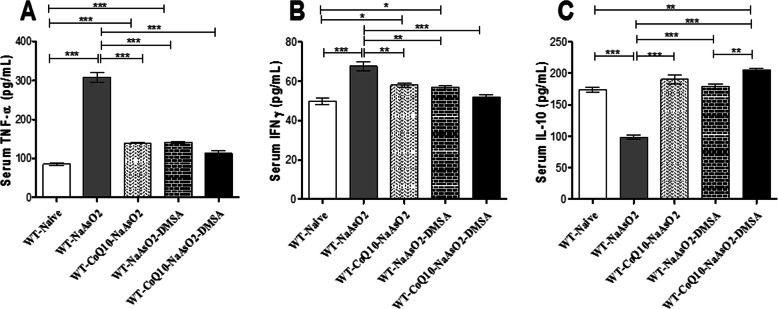
The figure shows the effect of arsenite, CoQ_10_ and DMSA on IFN-γ, TNF-α and IL-10. Male Swiss mice were treated with 15 mg/kg arsenite daily for 45 days. Two groups of mice were receiving 50 mg/kg DMSA or 200 mg/kg CoQ_10_ alone while another group of mice were treated using combination of the two. After the 45-day treatment period, mice were sacrificed, blood was drawn and serum was obtained and analyzed for cytokine levels using Sandwich ELISA. The bar graphs represent levels of serum IFN-γ (**a**) and TNF-α (**b**) and IL-10 (**c**). Data was analyzed using one-way ANOVA and Tukey’s post-hoc test. Bars represent ± SEM. The key for level of significance; *p < 0.05, **p < 0.01, ***p < 0.001. n = mice per group

Here, we report that exposure to sodium arsenite significantly (*p* < 0.001) elevated IFN-γ (Fig. 10a) and TNF-α (Fig. 10b) levels. Treatment with CoQ_10_ and DMSA singly or in combination protected from arsenite-driven elevation of pro-inflammatory cytokines (IFN-γ and TNF-α). Further, anti-inflammatory cytokine IL-10 (Fig. 10c) was significantly (*p* < 0.001) reduced in mice exposed to arsenite. Co-administration of CoQ_10_ and DMSA stabilized IL-10 levels; indicating positive modulation of arsenite-induced suppression of IL-10. Additionally, it was observed that when administered separately, CoQ_10_ and DMSA stabilised IL-10 levels in the presence of arsenic. There is statistical difference between the group on DMSA and the co-administered group (CoQ_10_ + DMSA) in the presence of arsenite. The elevation of IL-10 in the co-administered group, can only be attributable to the presence of CoQ_10_ based on the experimental design.

### Exposure to sodium arsenite deranged the pro and anti-inflammatory cytokine balance

The ratio between the pro- and anti-inflammatory cytokines was computed in order to establish whether the pro-inflammatory cytokines overwhelmed the anti-inflammatory responses and vice versa; and if administration of CoQ_10_ and DMSA alone or in combination mitigated the effects.

**Fig. 11:**
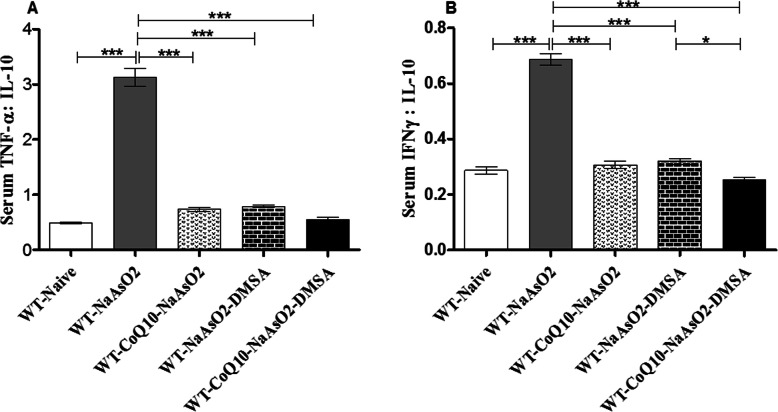
These figures are derived from the ratio between the pro and anti-inflammatory cytokines produced in the presence of arsenite, CoQ_10_ and DMSA. Mice were administered with 15 mg/kg arsenite_,_ 200 mg/kg CoQ_10_ and 50 mg/kg DMSA in the order indicated on the figure. Upon determination of the serum IFN-γ and TNF-α and IL-10 levels, TNF:IL-10 (**a**) and INF:IL-10 (**b**) ration were calculated and analyzed using one-way ANOVA and Tukey’s test for group comparison. Bars represent ± SEM. The level of significance: *p < 0.05, ***p < 0.001. *n* = 10

The ratio of TNF:IL-10 (Fig. 11a) and IFN-γ:IL-10 (Fig. 11b) in arsenite exposed mice was significantly (*p* < 0.001) elevated when compared to the control group. Administration of CoQ_10_ and DMSA alone or in combination normalized the balance between the pro and anti-inflammatory cytokines (Fig. 11 A, Fig. 11b).

### Histological analysis of the impact of arsenite, CoQ_10_ and DMSA on the liver, kidney and brain tissue

**Fig. 12:**
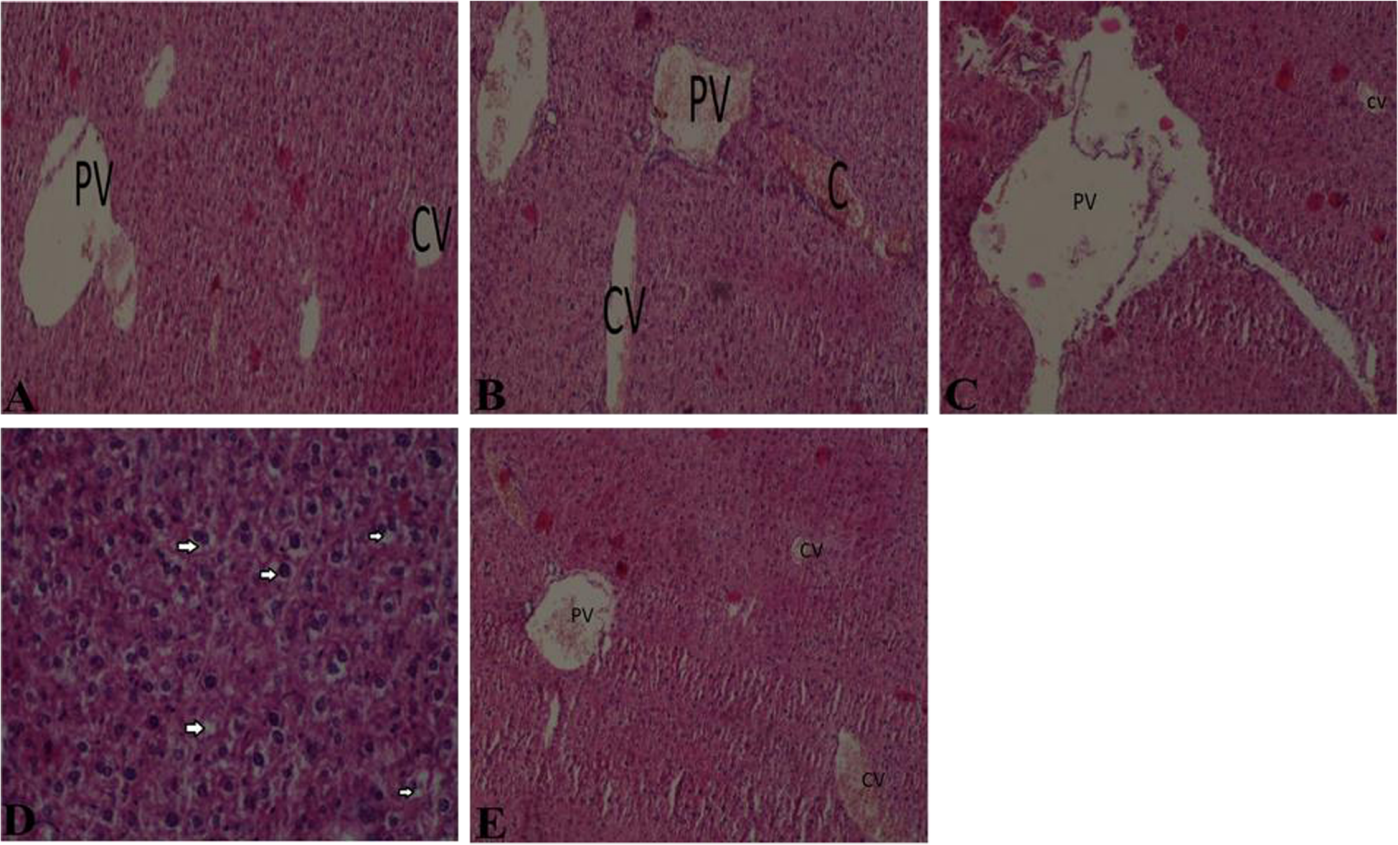
The figure shows the effect of arsenite, CoQ_10_ and DMSA on the liver histopathology. Mice were administered with 15 mg/kg arsenite alone or alongside 200 mg/kg of CoQ_10_ or 50 mg/kg DMSA independently or in combination. After 45-day post treatment, histological examination of liver tissue was conducted on the five groups; normal control (**a**), arsenite (**b**), arsenite+CoQ_10_ (**c**), arsenite+DMSA (**d**) and arsenite+ CoQ_10_ + DMSA (**e**). PV-portal vein, CV- central vein, C- congestion. Arrows indicate hepatocyte cytoplasm vacuolation

Consistent with enzyme/protein assays (Figs. 8 and 9), there was evidence for liver, kidney and brain damage on exposure to arsenite. Histological examination of the tissues was done to determine the extent of injury and protective role of CoQ_10_ and DMSA. In the liver, exposure to sodium arsenite had noticeable hepatic injury demonstrated by congestion of the hepatic vessels (Fig. 12b) in comparison with the control (Fig. 12a). Administration of CoQ_10_ assuaged the arsenite-induced hepatic damage (Fig. 12c). On the other hand, mice exposed to arsenite and treated with DMSA, demonstrated sub-acute liver injury characterized by hepatocyte cytoplasm vacuolation (Fig. 12d). Notably, co-administration of CoQ_10_ and DMSA resulted in abrogation of liver damage due to arsenite (Fig. 12e).

**Fig. 13:**
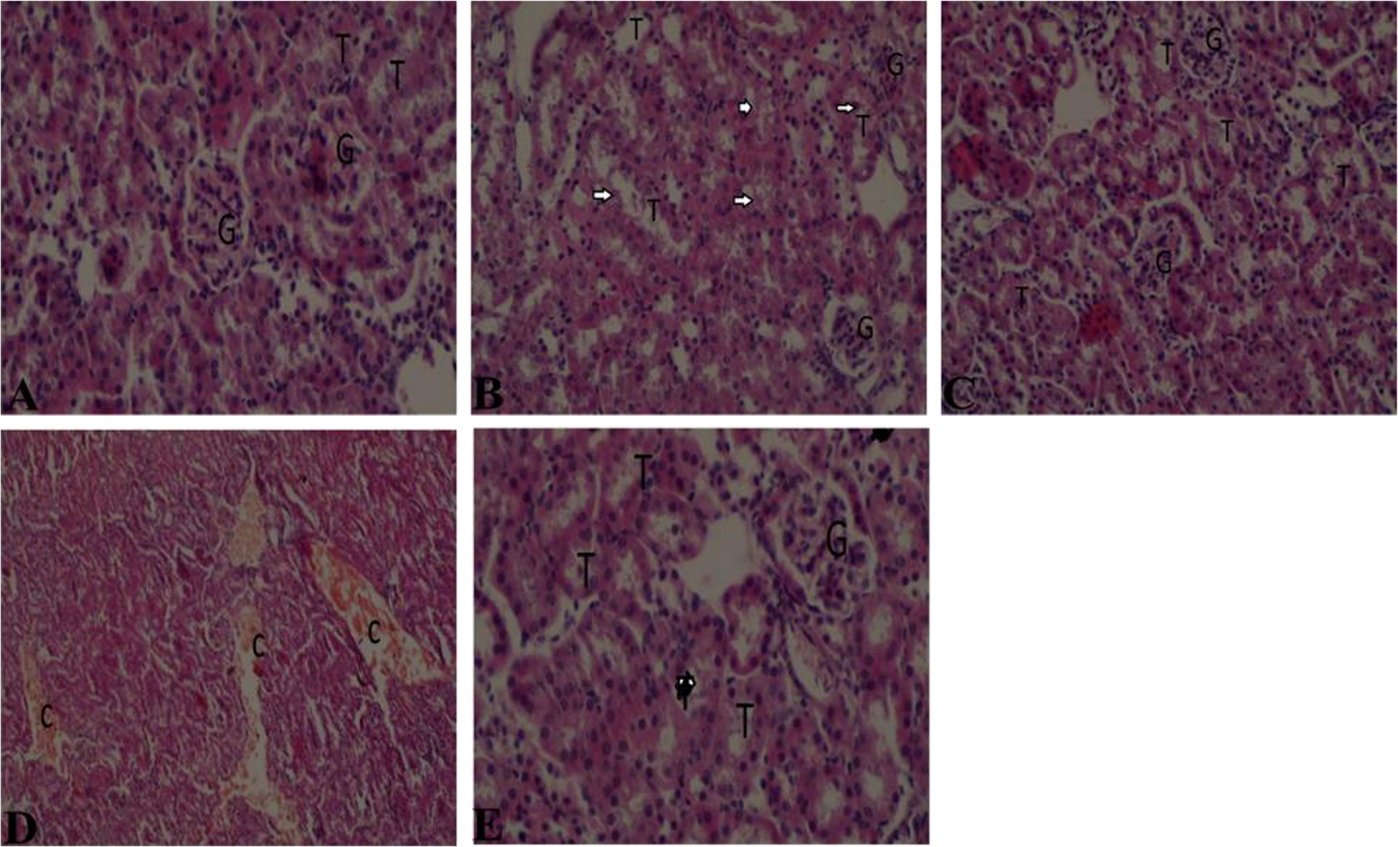
The figure shows the effects of arsenite on kidney tissues and the ameliorative effects of CoQ_10_ and DMSA. Mice were administered with 15 mg/kg arsenite alone or alongside 200 mg/kg of CoQ_10_ or 50 mg/kg DMSA independently or in combination. After 45 days’ post treatment, kidney samples from the five treatment groups; normal control (**a**), arsenite (**b**), arsenite+CoQ_10_ (**c**), arsenite+DMSA (**d**) and arsenite+CoQ_10_ + DMSA (**e**) were collected and processed for histological examination using H&E staining. G-glomerulus, T-renal tubules, C- congestion. Arrow show tubular cell vacuolation

Kidney histology also revealed tubular epithelium cell vacuolation (Fig. 13b) in mice exposed to arsenite, when compared with the control (Fig. 13a). Administration of CoQ_10_ to mice with prior exposure to arsenite resulted in recovery from arsenite-induced renal toxicity (Fig. 13c). However, DMSA did not have a beneficial effect on the arsenite-induced kidney injury (Fig. 13d). Co-administration of CoQ_10_ and DMSA fully protected mice from kidney injury due to arsenite exposure (Fig. 13e).

**Fig. 14:**
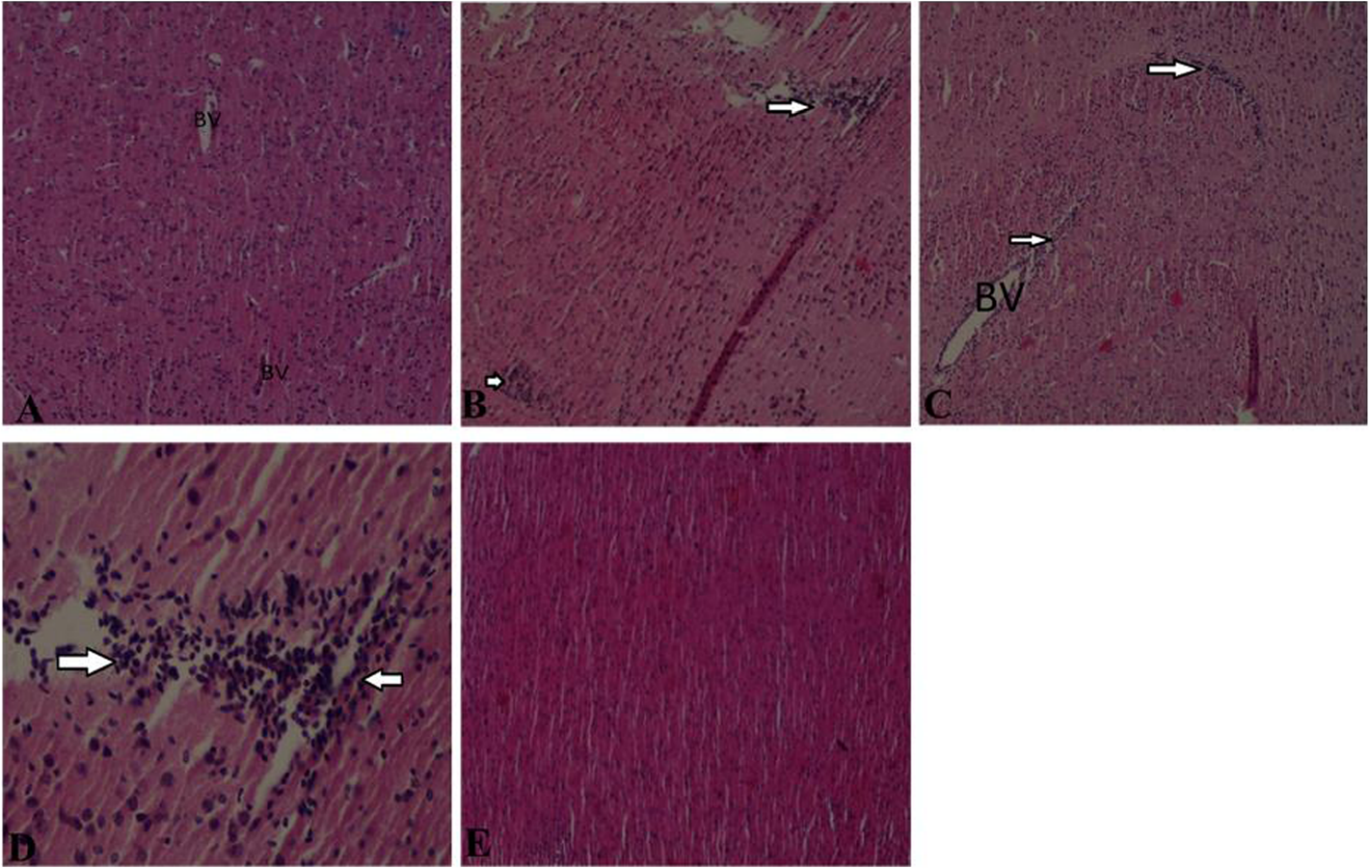
The figure shows the effect of arsenite on the brain and the neuroprotective role of CoQ_10_ and DMSA. Mice were administered with 15 mg/kg arsenite alone or alongside 200 mg/kg of CoQ_10_ or 50 mg/kg DMSA independently or in combination. After 45 days’ post treatment, brain samples from the five treatment groups; normal control (**a**), arsenite (**b**), arsenite+CoQ_10_ (**c**), arsenite+DMSA (**d**) and arsenite+CoQ_10_ + DMSA (**e**) were collected and processed for histological examination using H&E staining. BV-blood vessels, Arrows indicated areas of gliosis

Histological analysis of the brain tissue from the control group showed normal brain structural architecture (Fig. 14a). Mice exposed to arsenite showed brain injury characterized with focal areas of gliosis (Fig. 14b). Moreover, mice treated with CoQ_10_ showed a mild recovery from brain injury (Fig. 14c). Brain sections from the DMSA treated group were characterized with focal areas of gliosis, with regions of leukocyte aggregation (Fig. 14d). However, co-administration of CoQ_10_ and DMSA was neuroprotective from arsenite-induced neurotoxicity (Fig. 14e).

## Discussion

Exposure to various forms of arsenic cause serious pathologies that often result in debilitating illness and death. Current treatment utilizes 2,3-dimercaptosuccinic acid (DMSA), which works by chelation of extracellular arsenic. The chelation therapy does not get rid of the intracellular forms of arsenic, and therefore cellular damage persists. It therefore made sense to hypothesize that a strategy that chelates extracellular arsenic and protects from intracellular forms would produce a robust desired outcome. In this study, we report for the first time that CoQ_10_, a potent intracellularly acting anti-oxidant, enhanced the capacity of DMSA to protect from arsenite-induced cellular damage in a mouse model, simulating sub-chronic exposure. In a previous study, we reported the ability of CoQ_10_ to protect from organic forms of arsenic, in the brain [33]. In the current study, we demonstrate that when administered separately, and on occasion when co-administered with DMSA, assuaged arsenite-driven negative physiological and biochemical effects.

Oral exposure to arsenite evidently resulted in a significant reduction in mice body weight. The change in weight due to arsenic implies interference with growth and development; by impairing biochemical processes vital for normal growth [34]. Administration of DMSA significantly alleviated the arsenite-induced weight loss. Notably, CoQ_10_ did not have a significant impact on weight in the presence of arsenite due to its involvement in inhibition of adipocyte differentiation and lipid accumulation [35, 36].

Hematopoiesis is a very important process that guarantees efficient and regulated supply of a repertoire of blood cellular components [37]. In a healthy adult, approximately 10^11^–10^12^ new blood cells are generated every day to maintain steady state levels in the peripheral circulation [38]. Chemical toxins have been known to impair this process with serious health consequences. Maintenance of appropriate levels of RBCs is critical in ensuring that oxygen is delivered to all parts of the body in sufficient amounts; whereas WBCs are central to the desired function of the immune system, and healing. This study reports clear evidence for arsenite-driven impairment of hematopoiesis. There was an arsenite-induced decrease in the levels of the RBCs, hematocrit and hemoglobin; a clear indication of anemia. A similar observation was previously made by Ola-Davies and Akinrinde, [39]; where arsenite-induced generation of ROS resulted in lipid peroxidation of the RBC membrane and hemolysis. In addition, arsenic inhibits the erythropoietic process as well as heme synthesis [39, 40]. Interestingly, CoQ_10_ has shown robust ability to inhibit lipid peroxidation of the erythrocytic membranes [41]. In a related study by Thakur et al., [42], the potential of CoQ_10_ in assuaging sickle cell anemia due to its involvement in erythropoietin gene expression was observed. This finding strongly supports the ameliorative effect of CoQ_10_ in arsenic-induced anemia noted in this study. Notably, both DMSA and CoQ10 either alone or in combination, significantly ameliorated mice from arsenite-induced anaemia.

White Blood Cells (WBCs) play an essential role in immune response. Alteration of the total WBCs or its subtypes would have devastating consequences on immunity. In this study, exposure to arsenite resulted in elevation of the total WBC count, a clear indication of interference with the immune system by arsenic [39]. However, CoQ_10_ and DMSA maintained normal WBC levels in the presence of arsenite; essentially stabilizing WBC gene expression in the presence of the toxicant. Decreased levels of neutrophils, monocytes, and eosinophils provided additional evidence for arsenic-driven immune derangement. Arsenic-induced immunosuppression has the potential to predispose people to infections and in the long term, may contribute to microbial drug resistance [43]. The reduction of these cells through apoptotic induction has previously been demonstrated in patients treated with arsenic trioxide and in mice treated with sodium arsenite [44, 45]. It is noteworthy that arsenic trioxide (ATO); a trivalent arsenic, has been in use for the treatment of patients with acute promyelocytic anemia (ACA) due to its activity on malignant blood components through modulation of cell differentiation [46]. Prior studies have demonstrated that cytostatic and cytotoxic activity of arsenite trioxide in other cells masks the anti-differentiating activity against leukemic cells [47]. Besides, our study has demonstrated that sodium meta-arsenite; a trivalent arsenic, results in deleterious effects on hematopoietic cells, validating the fact that trivalent arsenics are cytotoxic not only by inhibiting erythroid differentiation, but also by inducing apoptosis by mitochondrial damage via activation of caspases 9,3 and 8 [45]. Additionally, this study has further demonstrated that treatment with CoQ_10_ alone or when combined with DMSA, is able to mitigate these adverse effects. It is therefore plausible that the anti-apoptotic activity of CoQ_10_ may be linked to the restoration of the observed arsenite-driven decrease in neutrophils, monocytes, eosinophils, and platelets as noted in the current study. Basophils constitute a type of white blood cells that play an important role in inflammatory responses. Therefore, the arsenite-induced elevation of basophils is perhaps indicative of active inflammation [48, 49]. The protective ability of CoQ10 against arsenite noted in this study, may be due to its proven potent anti-oxidant and anti-inflammatory properties [33].

Platelets, with other coagulation factors are essential in thrombosis and hemostasis. Platelets have been shown to participate in inflammatory response, microbial host defense and wound healing [50]. Consequently, alteration of platelets compromises these processes and poses a great risk for patients on blood thinning drugs. In the present study, exposure to arsenite significantly suppressed platelet levels; perhaps via the induction of caspase-dependent apoptosis through JNK activation [51], a clear indication of a greater risk for dangerous thrombotic events. Based on the comparison between the DMSA group and the co-administered group (CoQ_10_ + DMSA), CoQ10 protected from the DMSA-driven thrombocytosis in the presence of arsenic. Notably, DMSA appears to stimulate thrombocytosis, perhaps due to unintended side effects on vital molecules vital for regulating platelet levels. This is clearly important, suggesting a potential drug interaction by DMSA with drugs that modulate blood clotting cascades. This phenomena requires further scrutiny.

Normal lipid metabolism is very important in living organisms, including humans. In this study, arsenite appeared to interfere with lipid metabolism as demonstrated by elevation of cholesterol, triglycerides and high density lipoproteins. However, chelation of the circulatory arsenic and the ability of CoQ_10_ to regulate lipid metabolism and protect lipoproteins from oxidation provided a restorative effect as demonstrated by normalized lipid levels. Exposure to arsenite has previously been shown to increase hydrolysis of triglycerides resulting in release of free fatty acid (FFA) into plasma [52]. These events, including inhibition of lipoprotein lipase by arsenic results in hypertriglyceridemia and hypercholesterolemia [52].

The reduced form of glutathione (GSH) is a powerful antioxidant that also facilitates metabolism of xenobiotics such as drug and metal toxins. Additionally, it is utilized in the detoxification of methylglyoxal and formaldehyde, very toxic and harmful metabolites produced during active oxidative stress. Moreover, GSH is known to maintain vital exogenous antioxidants such as vitamins C and E in their reduced active states [53]. Note that the reduced form of glutathione is a key endogenous antioxidant that participates in scavenging of ROS [2]. Production of ROS due to the interconversions between pentavalent and trivalent forms during methylation process and conjugation of arsenic with glutathione for renal excretion is known to deplete GSH [54]. This study sought to determine the ability of CoQ_10_ and DMSA to protect from arsenite-driven GSH depletion and induction of oxidative stress. As expected, GSH was severely depleted in the liver and brain of mice exposed to arsenite. The significant depletion of liver GSH is due to its involvement in arsenic metabolism and elimination [55]. In the brain, arsenite-induced production of ROS that prompts cellular changes such as induction of apoptosis and necrosis of the neurons and astrocytes has been reported. The consequence is depletion of the endogenous antioxidants in the brain [56]. A slight increase in renal GSH was noted in mice administered with CoQ_10_ and DMSA; implying a recovery of the antioxidant system. Remarkably, the ability of CoQ_10_ to scavenge ROS and the chelating property of DMSA provided a robust response that abrogated arsenic-induced depletion of GSH, and consequently attenuated oxidative stress.

The liver plays a key role in the metabolism of xenobiotics and is the primary target for arsenic. Methylation and further metabolism of arsenite takes place in the liver. During arsenic poisoning, liver damage is inevitable. The liver is the primary target for arsenic due to the presence of high levels of methylation enzymes. High influx of arsenic molecules to the liver compromises the hepatocyte integrity [3]. In this study, liver function markers were measured to determine whether exposure to arsenite interfered with liver function, and if administration of CoQ10 and DMSA alone or in combination protected the liver from injury. There was an arsenite-induced elevation of serum AST and ALT. Note that under membrane stress, the levels normally increase and are released into the bloodstream, signaling hepatocellular injury [57]. CoQ_10_ and DMSA stabilized liver enzyme levels; implying a protective role in the presence of arsenite.

Bilirubin is a by-product of the metabolism of heme or heme-containing proteins such as cytochromes and caspases or from the rapid destruction of the erythroid within the bone marrow [58, 59]. The produced bilirubin is delivered to the liver for conjugation and is consequently excreted or recycled. Elevation of bilirubin and its accumulation in the liver results in inflammation and tissue damage [60]. Our results demonstrate that co-administration of CoQ_10_ and DMSA positively modulated arsenite-induced elevation of bilirubin; indicative of a beneficial role in arsenite-induced hepatocellular injury.

Gamma glutamyl transferase (GGT), is an enzyme utilized in the transfer of gamma glutamyl from compounds such as glutathione to an amino acid or peptide to form glutamate, an important excitatory neurotransmitter in the brain [61]. Additionally, GGT plays a key role in the synthesis and degradation of glutathione. The direct involvement of GGT in the maintenance of intracellular glutathione, makes it a reliable marker for oxidative stress, besides being indicative of liver damage when its serum levels rise significantly [62, 63]. Consequently, based on our findings, we conclude that elevation ALT, AST, GGT and bilirubin, signify significant arsenite-induced liver injury. Most significantly, co-administration of CoQ_10_ and DMSA abrogated arsenic-induced alteration of respective enzymes/protein levels.

Further investigations focused on the integrity of kidney function in the presence of arsenite, CoQ_10_ and DMSA. Disposition of arsenic in renal tissue facilitates methylation to toxic metabolites that cause inflammation triggering the TNF-α mediated apoptosis [64]. These events and the increased renal excretion of the metalloid may be associated with the kidney injury that was observed in this study as demonstrated by the elevation of serum creatinine levels. We demonstrate that arsenic-induced renal injury was assuaged by co-administration of CoQ10 and DMSA; implying an ameliorative role. The results indicate that inorganic arsenic can cause kidney damage, and most importantly, CoQ10 and DMSA protect the kidney from such injury.

Exposure to arsenic has been shown to activate the inflammasomes which in turn, induces the production of pro-inflammatory cytokines like TNF-α and IFN-γ [65, 66]. Furthermore, exposure to arsenic activates NF-κB expression with concomitant expression of elevated levels of pro-inflammatory cytokines TNF-α, and IL-6 that are associated with the inflammatory process [67, 68]. In the current study, exposure to arsenite resulted in an increase of TNF-α and IFN-γ, indicative of arsenite-driven inflammation. Our study revealed that treatment with DMSA/CoQ_10_ alone or in combination ameliorated the arsenic-induced augmentation in murine serum TNF-α and IFN-γ. This results corroborates our early work on the anti-inflammatory ability of CoQ_10_ on NF-kB activation [33].

The ratio between anti-inflammatory and pro-inflammatory cytokines determines the inflammatory status [69]. Furthermore, we show that the altered levels of serum anti-inflammatory cytokine IL-10 reflects aggravated inflammation from arsenic toxicity (Fig. 10c). Particularly, depleted serum levels of immunologically important IL-10 in arsenite-exposed groups of mice is a clear indication of an inflammatory response to arsenic toxicity. There was prominent imbalance of pro-inflammatory and anti-inflammatory cytokines in arsenite-exposed group of mice that may lead to exacerbation of inflammation process. Of particular interest is the achieved balance of pro-inflammatory and anti-inflammatory cytokines when CoQ_10_ and DMSA were administered separately or together. This beneficial effect can be attributed to the chelation properties of DMSA and the anti-inflammatory properties of CoQ_10_ [33, 70, 71]. These results clearly demonstrate that CoQ_10_ and DMSA modulate the immune system in a manner that may attenuate arsenic toxicity driven by inflammation.

Production and accumulation of ROS during arsenic metabolism is associated with organ toxicities. Using standard histopathological analysis, this study determined the negative effects of arsenite on liver, brain and kidney; as well the ability of CoQ_10_ and DMSA to protect from the arsenite-induced organ damage. As expected, exposure to sodium arsenite resulted in liver, brain, and kidney injury characterized by hepatocyte vacuolation, gliosis, and renal tubular congestion respectively (Fig. 12 b, Fig. 13 b and Fig. 14 b). Tissue examination of liver, brain, and kidney revealed the role of CoQ_10_ and DMSA in respective organ protection from arsenic toxicity. In all tissues examined via histology, it was evident that DMSA did not provide the desired protection from arsenite toxicity. This can be attributed to the inability of DMSA to be well distributed in intracellular spaces due to its lipophobic and hydrophilic properties [72]. Notably, CoQ_10_ independently and when combined with DMSA, assuaged arsenic-induced tissue injury as per the histology results. The ability of CoQ_10_ to be well distributed in extracellular and intracellular spaces is critical in its effectiveness against arsenic toxicity [73]. Based on these findings, and with further studies, it is evident that a new formulation that utilizes both CoQ_10_ and DMSA provides an excellent opportunity and great promise to prevent and treat arsenite-induced toxicity and organ damage.

## Conclusion

Overall, findings from this study clearly demonstrate the deleterious effects of oral exposure to sodium arsenite and ability of CoQ_10_ and DMSA to protect from such. Certainly, these new observations undeniably present unique promising opportunities for further studies to elucidate how CoQ_10_ may be applied as an adjunct therapy to prevent or support recovery from arsenic poisoning both at acute and chronic levels.

## Data Availability

All data has been made available. Additional requests for data will be complied with as appropriate.

## Abbreviations

DMSA: 2,3-dimecaptosuccinic acid
CoQ10: Coenzyme Q 10
GSH: Glutathione
ROS: Reactive Oxygen Species
RMCB: Rapid Murine Coma Behavioural Score
SOD1: Superoxide Dismutase 1 (Cu-Zn)
WHO: World Health Organization
MMA^III^: Monomethylarsonous Acid
DMAA^III^: Dimethylarsinic acid
BAL: Dimercaprol
DMPS: 2,3 Dimercaptopropanesulphonate sodium
DMSA: Meso-2,3-dimercaptosuccinic Acid
TMAO: Trimethylarsine Oxide
SAM: S-adenosylmethionine
ALT: Alanine aminotransferase
AST: Aspartate aminotransferase
GGT: Gamma-glutamyl transferase
NADPH: Reduced nicotinamide adenine dinucleotide phosphate
DNTB: 5-(2-aminoethyl) Dithio-2-nitrobenzoic acid
TNFα: Tumour necrosis factor alpha
IFNγ: Interferon gamma
IL-10: Interleukin 10
RBCs: Red blood cells
WBCs: White blood cells
